# Cases from the Surgical Clinic of Prof. Moses Gunn, of Rush Med. College

**Published:** 1870-12

**Authors:** H. F. Chesbrough


ARTICLE VIII.—*Cases from the Surgical Clinic of Professor Moses Gunn, of Rush Medical College.* Reported by H. F. CHESBROUGH, M.D.

[Continued.]

X. P. P., a young lad of sixteen, who had been before the class many times during the previous year for treatment of hip-joint disease in the suppurative stage, came to the clinic on the first Saturday in October, for resection of the head of the femur. When the lad first came under observation, his hip was suppurating extensively. Still, as cod liver oil and iodide of potassium agreed with him—as his appetite remained good, and the boy was not losing ground, no operative procedure was deemed necessary.

But at last the patient evidently began to lose flesh and appetite, and to show signs of being overcome by the severity of the disease. Now hope of a cure by anchylosis was abandoned, and all reliance placed in an exsection as the only chance of saving the patient's life.

Dr. Freer was present, and assisted in the operation. When the joint had been laid bare by a T-shaped incision, and the capsule freely laid open, an assistant used the leg as a lever to force the head of the femur out of the acetabulum, and the bone fractured just below the lesser trochanter. This was considered a very unfortunate accident at the time, but on examining the fragment after it had been removed, it was found to be carious at the base of the lesser trochanter, as well as in the head and neck. The lamellar structure of the shaft was very thin and weak, which accounted for the ease with which it fractured.

The wound was dressed with a pledget of lint, washed out with a solution of carbolic acid, and moderate extension and counter-extension maintained. The case progressed favorably, and on Saturday, Nov. 12th, 1870, he again appeared in the clinic, walking readily with a crutch, and the wound entirely healed. Up to this time he had not been allowed to walk without a crutch. It is evident now that he will soon be able to walk with merely a cane, and that the operation has not only saved his life but also given him a very useful limb.

XI. On the evening before the "Fourth" (July, 1869), Mrs. M. and her husband had a little discussion while under the influence of mountain dew. The next morning she discovered that her left shoulder was lame and painful, and procured some liniment for it. After about nine weeks she concluded that the shoulder did not get well quite fast enough, and applied to the Dispensary for relief. She was told that her arm was dislocated, and to come again on Saturday. About three weeks after this, she put in an appearance on Saturday (Oct. —). After she had been fully anæsthetized, the adhesions which the head of the bone had formed with the tissues about it were first forcibly and thoroughly broken up. The arm was then raised up by the side of the head, and extension by two assistants was made, while Professor Gunn fixed the shoulder with both hands, and with the tips of the fingers followed the head of the humerus as it retreated upward from the axilla. When the head of the bone was sufficiently elevated, he endeavored to maintain it in position, while the assistants, still keeping up extension, gradually swept the arm downward till it rested by the side of the body. The third effort was successful. The arm was then put in a sling, and bound up securely in its place by the roller bandage.

The woman has since been back to the College, and pronounces her arm as good as ever.

XII. December 18, 1869, brought another case of downward dislocation of the right humerus. This case was of six weeks standing. The patient was treated in a similar manner, and at the first trial (the dislocation being much more recent) the luxation was reduced.

The arm was fixed by a roller, and worn in a sling, and was a perfectly serviceable member in a very few weeks.

